# Supplementary material for: Effects of Nutrition on Cognitive Function in Adults with or without Cognitive Impairment: A Systematic Review of Randomized Controlled Clinical Trials
Source: Nutrients. 2021 Oct 22;13(11):3728. doi: 10.3390/nu13113728 (PMC8621754; doi:10.3390/nu13113728)
Supplement: Supplementary file 1 [file nutrients-13-03728-s001.zip › nutrients-1413032-supplementary.pdf]

Supplementary Files

Figure S1. Pubmed search strategy.

((nutrients OR food OR nuts OR cereals OR vegetables OR fruit OR fish OR legumes OR milk OR yogurt OR cheese OR seeds OR dairy product OR juice OR berry OR berries OR fiber OR olive OR diet OR dietary OR supplementation) AND (Alzheimer's disease OR cognitive impairment OR dementia) AND ((randomizedcontrolledtrial[Filter]) AND (humans[Filter]) AND (english[Filter]))) AND (("2018/01/01"[Date - Create] : "2021/07/31"[Date - Create]))

Figure S2. Quality assessment of the RCTs that followed an intention-to-treat analysis.

Study	Risk of bias domains					
	D1	D2	D3	D4	D5	Overall
Abe S., 2020	+	+	+	X	+	X
Ahles S., 2020	+	+	+	X	+	X
Baleztena J., 2018	+	+	+	+	+	+
Ban S., 2018	-	+	+	+	+	-
Bensalem J., 2019	-	+	+	+	+	-
Blumenthal J., 2019	+	+	+	+	+	+
Chhetri J., 2018	+	+	+	+	+	+
Delrieu J., 2020	+	+	+	+	+	+
Edwards C., 2020	-	+	+	+	+	-
Fukuda T., 2020	+	+	+	+	+	+
Giudici K., 2020	+	+	+	+	+	+
Herrlinger K., 2018	-	+	+	+	+	-
Hu J., 2018	+	+	+	X	+	X
Hwang Y., 2019	+	+	+	+	+	+
Komulainen P., 2021	+	+	+	+	+	+
Li M., 2021	+	+	+	+	+	+
Ma F., 2019	-	+	+	X	+	X
Marriott B., 2021	+	+	+	+	+	+
Owusu J., 2019	+	+	+	+	+	+
Sala-Vila A., 2020	-	-	+	+	+	-
Wade A., 2019	-	+	+	X	+	X
Wade A., 2020	-	+	+	X	+	X
Yuda N., 2020	+	+	+	+	+	+
Zajac I., 2020	+	+	+	+	+	+

Domains:
D1: Bias arising from the randomization process.
D2: Bias due to deviations from intended intervention.
D3: Bias due to missing outcome data.
D4: Bias in measurement of the outcome.
D5: Bias in selection of the reported result.

Judgement
X High
- Some concerns
+ Low

Figure S3. Quality assessment of the RCTs that followed a per-protocol analysis.

Risk of bias domains

Study	D1	D2	D3	D4	D5	Overall
Arellanes I., 2020	+	+	+	+	+	+
Boyle N., 2019	+	+	+	+	+	+
Carmichael O., 2018	-	+	+	+	+	-
Chai S., 2019	+	+	+	+	+	+
Chupel M., 2018	-	+	+	X	+	X
Cox K., 2020	+	+	+	+	+	+
Danthiir V., 2018	+	+	+	+	+	+
Decroix L., 2019	+	+	+	+	+	+
Fukuda T., 2020 [2]	+	+	+	+	+	+
Hamasaki A., 2019	+	+	+	+	+	+
Igase M., 2018	+	X	+	+	+	X
Ito N., 2018	+	X	+	+	+	X
Kita M., 2018	+	+	+	+	+	+
Kuroda Y., 2019	-	+	+	X	+	X
Kuszewski J., 2020	+	+	+	+	+	+
Lau H., 2020	+	X	+	+	+	X
Masuoka N., 2019	-	X	+	+	+	X
McNamara R., 2018	-	+	-	+	+	-
Ochiai R., 2019	+	+	+	+	+	+
Perry N., 2018	-	+	+	X	+	X
Reid S., 2018	+	X	+	+	+	X
Saitsu Y., 2019	+	+	+	+	+	+
Sakurai K., 2020	-	X	+	+	+	X
Sandberg J., 2018	-	X	+	X	+	X
Schönhoff M., 2018	-	X	+	+	+	-
Schwarz C., 2018	+	X	+	+	+	X
Stavrinou P., 2020	+	X	+	+	+	X
Suzuki T., 2019	-	X	+	X	+	X
Tabue-Teguo M., 2018	+	+	+	+	+	+
Tadokoro K., 2019	-	X	X	X	+	X
Tohda C., 2020	+	X	+	+	+	X
Tsolaki M., 2020	+	+	+	+	+	+
Uenobe M., 2019	-	X	X	X	+	X
Varanoske A., 2018	+	+	+	+	+	+
Wirth M., 2018	+	X	+	+	+	X
You Y., 2021	+	+	+	+	+	+
Zhu X., 2020	+	+	X	+	+	X

Domains:
D1: Bias arising from the randomization process.
D2: Bias due to deviations from intended intervention.
D3: Bias due to missing outcome data.
D4: Bias in measurement of the outcome.
D5: Bias in selection of the reported result.

Judgement
X High
- Some concerns
+ Low
